# Clinician perceptions of factors influencing referrals to a smoking cessation program

**DOI:** 10.1186/1471-2296-9-18

**Published:** 2008-03-28

**Authors:** Jodi Summers Holtrop, Rebecca Malouin, David Weismantel, William C Wadland

**Affiliations:** 1 Department of Family Medicine, Michigan State University, B101 Clinical Center, East Lansing MI 48824, USA

## Abstract

**Background:**

Referral of patients to smoking cessation telephone counseling (i.e., quitline) is an underutilized resource by primary care physicians. Previously, we conducted a randomized trial to determine the effectiveness of benchmarked feedback on clinician referrals to a quitline. Subsequently, we sought to understand the successful practices used by the high-referring clinicians, and the perceptions of the barriers of referring patients to a quitline among both high and non-referring clinicians in the trial.

**Methods:**

We conducted a qualitative sub-study with subjects from the randomized trial, comparing high- and non-referring clinicians. Structured interviews were conducted and two investigators employed a thematic analysis of the transcribed data. Themes and included categories were organized into a thematic framework to represent the main response sets.

**Results:**

As compared to non-referring clinicians, high-referring clinicians more often reported use of the quitline as a primary source of referral, an appreciation of the quitline as an additional resource, reduced barriers to use of the quitline referral process, and a greater personal motivation related to tobacco cessation. Time and competing demands were critical barriers to initiating smoking cessation treatment with patients for all clinicians. Clinicians reported that having one referral source, a referral coordinator, and reimbursement for tobacco counseling (as a billable code) would aid referral.

**Conclusion:**

Further research is needed to test the effectiveness of new approaches in improving the connection of patients with smoking cessation resources.

**Trial Registration Number:**

Clinicaltrials.gov NCT00529256

## Background

Tobacco use continues to be the number one cause of preventable death in the United States [1,2]. Primary care clinicians play an important role in the identification, assessment and treatment of tobacco addiction. The Clinical Practice Guideline, Treating Tobacco Use and Dependence, recommends that clinicians use the 5 A's: *Ask *if the patient uses tobacco, *Advise *tobacco using patients to quit, *Assess *patient willingness to quit, *Assist *patients who are willing to quit or provide a motivational message to those unwilling to quit, and *Arrange *follow-up [3].

Although implementing this clinical practice guideline has been shown to be effective in reducing smoking in primary care patients and is one of the most cost-effective primary care interventions available, many clinicians do not regularly utilize it [4-9]. The National Ambulatory Medical Care Survey, an annual survey of a random sample of US office-based physicians, found that 32% of patient charts did not include information about tobacco use, 81% of smokers did not receive assistance and less than 2% received a prescription for pharmacotherapy [10]. Other studies have also found low rates of tobacco intervention [11,12]. Further research has found that clinicians are especially lacking in providing assistance and referral (arrange) to additional services, even though many states offer quitline counseling as a free service and it appears to be feasible to do so [13,14]. Surveys of clinicians reveal many barriers including lack of time, lack of reimbursement, perceived patient resistance, lack of provider confidence and/or training, knowledge of benefits of physician intervention, and limited resources to assist smokers [15-17]. A more specific understanding of these barriers and how they may be overcome is needed.

Our research team conducted a randomized controlled trial to investigate the influence of benchmarked feedback on referrals to a quitline and found that specific feedback significantly influenced greater referrals in the intervention (feedback) than control (no feedback) group [18]. However, across groups, some clinicians referred at a high rate and some had no referrals. Although the quantitative analyses provided clues as to why some clinicians referred more than others, we sought a greater understanding of how the clinicians operationalized the 5 A's in their practice and how this related to higher or lower referral rates to the quitline service. Thus, we performed a qualitative sub-study, described in this paper, using thematic analysis, to explore contributors to effective practices for high-referring clinicians; and barriers, motivating factors, suggestions for improving referral services, and use of incentives for both high and non-referring clinicians.

## Methods

The study was approved by the University Committee for Research on Human Subjects at Michigan State University and all institutional review boards (IRB's) associated with the participating practices (total of 26 unique IRB's).

### Randomized Trial

This sub-study sampled clinicians participating in a randomized trial [18] Briefly, the purpose of the trial was to determine if benchmarked feedback to clinicians on their referrals to a quitline influenced their referral rate, over a control (no feedback) condition. Clinicians were asked to refer smoking patients by handing patients cards to call for quitline participation, or by the medical practice faxing a patient referral to the quitline. Referrals were made to one specific quitline, which was owned and managed by the major health insurer in the state. The quitline agreed to accept all referrals from study clinicians, regardless of patient insurance type, during the course of the study. The results indicated a significant difference in the intervention group having an overall greater number of referrals than the control group, however, the majority of referrals were due to specific high-referring clinicians.

### Subjects

Eligible subjects included the clinicians (physicians, nurse practitioners, and physician's assistants) in the feedback trial [18]. We compared two groups of clinicians within this larger subject pool (n = 308) regarding how many referrals were made over the course of the study to the specified quitline: high-referring (top 10% of all referrals; n = 28) and non-referring (zero referrals; n = 195). All of the 28 high-referring clinicians were invited for participation. Of the 195 non-referring clinicians, we sought to represent characteristics of the high-referring clinicians. Therefore, we purposefully selected non-referring clinicians. First, we listed all practices that included a non-referring clinician. Then, we identified those practices listed that were matched to the high-referring clinician practices with regard to allocation status (intervention or control), size of practice, and region of the state. To avoid a biased selection of clinicians, once the practices were selected, we then randomly ordered the clinicians in each practice and invited the first clinician listed for interview. If the first declined, we invited the second listed and so on. Generally, these clinicians were found in practices that did not include high-referring clinicians. In a small number of cases, non-referring clinicians were in the same practice as a high-referring clinician. In this case, the high-referring clinician was not included in our random ordering.

### Recruitment and Interview Procedures

Identified clinicians were invited to participate by written invitation letter, then a follow-up telephone call. Up to six call attempts were made to confirm participation with an interview, which was scheduled at the convenience of the clinician. After six attempts or refusal, we moved on to the next clinician. Verbal consent was obtained at the beginning of the taped interview. The interviews were conducted by a master's degree prepared nurse educator trained by a qualitatively-trained investigator. Clinicians were compensated $25, and interviews were conducted between October, 2004 and March, 2005.

In order to elicit perceptions from busy clinicians in disperse geographic locations within a specific timeframe, we developed a semi-structured interview guide (appended). All interviews with high-referring clinicians were conducted until all clinicians willing to be interviewed were completed. Interviews with additional non-referring clinicians were continued until the investigators determined that clinician perceptions began to repeat themselves, cuing the investigators that saturation or the range of responses had been achieved.

### Data Analysis

To provide a more nuanced understanding and interpretation of the textual data, two trained qualitative researchers coded the transcript data. One was internal and one external to the project. The external researcher had extensive discussions with the study team to understand the objectives of the overall study and sub-study. Investigators utilized a thematic analysis of the data, whereby themes are identified in the textual data [19]. Investigators included a priori themes, derived from the research and, consequently, interview questions, and emergent themes or concepts. Interview data were transcribed directly from the tape recordings. Individual phrases within respondent interviews were categorized using open coding. Each phrase was then coded according to a theme using selective coding. Investigators first independently coded each interview. Both coded all interviews independently, then together. Then, an iterative process was utilized as the investigators compared coded interviews and came to common agreement on categories, developed additional questions or probes based on initial interviews, and re-analyzed the data to develop minor categories, and organize the data into major categories, which were then organized into a priori and emergent themes. Examples of a priori themes include barriers to and benefits of referrals to cessation programs and aspects of the 5 A's. Examples of emergent themes include motivation, particularly in reference to patient motivation. Minor categories represented specific subsets of the major categories. Themes and included categories were then organized into a thematic framework. The investigators then shared the completed analytic results with a sub-group of the clinicians for member-checking of main themes.

## Results

### Subjects

**Figure 1:**
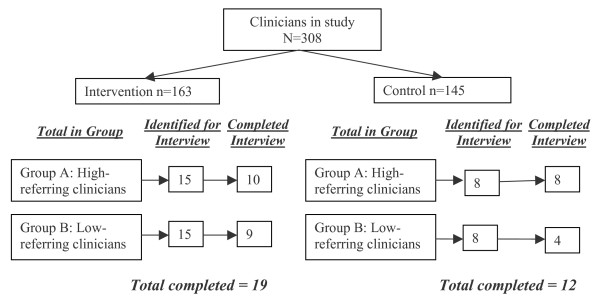
Subject Flow.

Thirty-one clinicians from 21 practices participated in the interviews. Eighteen clinicians were from the high-referring clinicians and 13 from the non-referring clinicians. Figure 1 demonstrates the response rate to the request for interviews. Inability to establish an interview time was the primary reason for non-completion.

Characteristics of participating clinicians are included in Table 1. The practices represented a variety of practice characteristics including primary care practice type (family medicine, general internal medicine, and obstetrics/gynecology), size (small to large), geographic location (rural, urban and suburban) and varying patient populations, including high and low smoking rates, health insurance coverage, and racial/ethnic diversity.

**Table 1:** Participating Clinician Characteristics

	**High-Referring**	**Non-Referring**
Gender		
Female	9 (50%)	4 (31%)
Male	9 (50%)	9 (69%)
Graduated Residency		
Before 1990	11 (61%)	6 (46%)
1990 or After	6 (33%)	7 (54%)
Unknown	1 (6%)	0
Specialty		
Family Medicine	9 (50%)	8 (62%)
Internal Medicine	5 (28%)	2 (15%)
Obstetrics/Gynecology	4 (22%)	3 (23%)
Clinician Type		
Nurse Practitioner	2 (11%)	1(8%)
Physician Assistant	1 (6%)	0
Physician	15 (83%)	12 (92%)
Patients Per Day		
Mean (Standard Deviation)	19.9 (8.05)	21.4 (7.5)
Estimated Smokers Per Day		
Mean (Standard Deviation)	2.4 (1.14)	2.9 (1.38)

### Main Themes

Table 2 outlines the themes and major and minor categories. The themes are described below and include particularly salient quotes from clinician participants, which are noted by group (high or non-referring). Any differences between high and non-referring clinicians regarding any of the thematic areas is also noted and described. Overall, there were few differences between high and non-referring clinicians.

**Table 2:** Themes, Major and Minor Categories

Theme	Major Category	Minor Category
Procedure	Ask/Identification	forms (*flowsheet, encounter form, chart intake, history, problem list*)
		process (*initial interview, annual physical, for chronic disease or respiratory infection*)
		staff (*nurse, physician*)
Procedure	Assessment	interest in quitting
		risk of disease
		habits
Procedure	Advise/Assistance/Arrange	stage of change
		pharmacotherapy
		Counseling
		Brochures
		Programs
		Follow-up visit
Barrier	Provider	Provider Characteristics (*forgetfulness, program knowledge, fear of "badgering" patien*t)
		Practice characteristics (*type, staff turnover, lack of billing code*)
		Patient characteristics (*desire to quit, insurance type, addiction*)
Benefit	Quit line Program	Single source for referral
		Personalized attention
Barrier	Quit line Program	Provider Characteristics (*lack of reminders, lack of feedback, only for one type of insurance, lack of advertising*)
		Patient Characteristics (*comfort with program, short term solution, insurance type*)
Procedure	Satisfaction	Good (*identification, assessment, advise*)
		Poor (*assistance, arrangement, follow-up*)
Motivation	Provider	Individual (*family death, concern for own health, patient care*)
		Program (*ease, one referral, feedback*)
		General (*healthcare costs, good medicine*)
Motivation	Patient	Cost
		Support/feedback
Incentives	Provider	Physician responsibility
		Code for patient education/prevention

#### Use of the 5 A's for tobacco treatment

##### Satisfaction with process of the 5 A's

Across both groups, clinicians reported being satisfied with their process for identification of tobacco use, but not with assistance, or follow-up (arrange).

"I'm very satisfied with the way I do the screening for smokers and talking about quitting, but our follow up is poor...I am not satisfied with that at all." (non-referring)

High-referring clinicians reliance on the quitline: High-referring clinicians relied on the quitline as a primary source of referral, whereas non-referring clinicians reported doing their own counseling without referral, having a variety of resources, or did not provide treatment beyond brief advice and pharmacotherapy. The satisfaction with the clinician's self-selected process did not vary by groups. The high-referring clinicians perceived fewer barriers to using the quitline process including fax forms and patient handout cards and reported using them more often.

"... the basic interview and treatment program and the referral – I think I have very good success in this procedure. I don't have any problems with it." (high-referring)

#### Barriers to referring patients to cessation services

##### Time to intervene

Across groups, the main barrier to tobacco cessation intervention was the time it took and competing priorities for that time.

"... just in general time. I mean, we're under enormous pressure to see more patients in less time. And unfortunately, it does take time to do this counseling. I tend to try to take it anyway, but of course that puts me behind and that [the insurance company] also rates me based on patient satisfaction with how on schedule I am, so really I'm damned if I do, and damned if I don't." (high-referring)

"...and although I think that the smoking cessation program that [the insurance company] offers could be an excellent program, its just – it gets lost in the shuffle" (non-referring)

##### Multiple referral sources

A specific barrier to initiating referral was the presence of multiple places for referral of smokers. This appeared to be more of a barrier for clinicians not referring to the quitline.

"We've got one insurance, who's eligible, who's not eligible, where the hell are the little pamphlets at..." (non-referring)

"... you know I'll find out if they're smoking usually when I'm in the room talking with them and it's a barrier to run out to find out well what's the insurance company that (has) this program and (which) one (has) that program and it takes too much time and too much effort to do things that way." (non-referring)

#### Motivations for referring patients to services

##### Personal importance

Although all groups were motivated to refer patients to services, those in the high-referring group noted a stronger feeling of importance to tobacco cessation and several cited personal reasons for this importance.

"...it's a matter of being comprehensive in health care...we'd all be a lot healthier. And I'd feel a lot healthier... " (high-referring)

"I want them to not be smoking. And I desperately want this for my patients; on a personal side, my parents both died lung cancer deaths. So this is something that truly is important to me and has been for a long time." (high-referring)

##### Patient responses

Across groups, patient responses to suggesting cessation referral often motivated clinicians to either refer more or less. Encouraging factors across groups included reports of the satisfaction of seeing patients quit and patients reporting satisfaction with the quitline service. Discouraging factors included reports of patients not wanting someone to call them, concern for "badgering," and the cost of cessation related medications or services.

"My level of satisfaction is high when they do kick, I get a big kick out of that and one thing that I do with patients is I ask them to bring me their past pack of cigarettes and then I make a trophy out of it and tape it to a sheet of paper and paste it up on the wall." (non-referring)

"... when people were like, "Oh no, I don't want anyone to call me. I think that they have a misperception – they felt like somebody was going to be calling them on the phone nagging them and lecturing them." (high-referring)

"I have a problem with people who say they can't take the Zyban because it costs too much when it costs less than a pack of cigarettes..." (non-referring)

#### Clinician suggestions for improvement of referral services

##### Interest in patient-specific feedback

Clinicians reported an interest in patient-specific (if their patient quit) in addition to general (how many referrals were made) feedback, which they reported as a suggestion for motivating future referrals. Some clinicians in the intervention group reported the feedback reports as motivating them to refer patients by keeping their attention on the topic.

"...I have a lot going on with the patients ... other than smoking, if there's a program in place that can actually track whether or not the patient is successful in quitting really is what I'm most interested in is maybe six month follow-up after they've called saying yes the patient quit or no the patient did not quit, even providing their name, that is something I can put in the chart..." (non-referring)

"I want to know how many people that we enrolled quit. And successfully quit after one year." (high-referring)

Assistance from outside the practice: The idea of having help from a service outside of the practice for patient referral was suggested. Busy clinicians lack the time of themselves or their staff to do a thorough job with tobacco counseling and tracking progress.

"Just having, you know, someone other than me involved...Because, you know, you can really turn patients off if you nag them too much, and bringing in another person, you know, is helpful for that reason." (high-referring)

##### Coordination assistance

Another suggestion included assistance in the form of a person to coordinate referrals. None of the clinicians reported having such a person in his/her practice.

"...if we had a coordinator who would be given the information, collated it, and sliced and diced it, and presented it at a provider meeting, that probably would help." (non-referring)

"...and just remembering to do it, you know ... maybe have somebody, like a university or an independent contractor stop by the office and make sure that they have the supplies and keep doing it, that would make it easier." (high-referring)

##### One referral resource

Clinicians reported a desire to improve referrals by having one place to refer all patients. Those successful in referring patients were clinicians that had a trusted referral resource and used it often, rather than trying to manage referral to all the different options.

"...it's wonderful to have a single referral source that I can simply refer people to, and it make the job infinitely simpler. And it actually makes it possible in my mind. It's almost impossible if within the context of our office we have to look up and see what the health plan is, and then try to match that against the appropriate referral capabilities." (high-referring)

"... That's what I'm looking for – the single number and the one-size fits all, from my standpoint, from the referring physician standpoint" (high-referring)

#### Payment and Incentives

Finally, clinicians were asked about financial motivators for increasing quitline referrals. Overwhelmingly clinicians endorsed reimbursement as a patient visit. In general, clinicians did not think that payment for number of referrals generated was appropriate and there was a mixed response to pay for performance incentives.

"I think providing – getting paid for what you actually do – you know, a lot of physicians – feel like we're doing all this stuff ... and never get paid for it..." (non-referring)

". I want the diagnosis and a code so that when this patient comes in for nothing else except smoking cessation that I can bill that code and get paid for it." (high-referring)

"I think if there isn't an incentive for a certain outcome or there are multiple competing agendas that the things that are incentivised are the things that are gonna get done." (non-referring)

## Discussion

The clinicians in this sub-study were participants in a larger trial examining response to specific benchmarked feedback on their referrals to a quitline. In this qualitative sub-study, we found very similar responses in both high-referring and non-referring clinicians regarding the barriers to using the 5 A's and referring patients to quitline services. Differences were that high-referring clinicians in both the intervention and control groups of the feedback trial used the quitline as a method of offering referral to smoking patients, whereas non-referring clinicians did not describe consistency in methods of referral. The high-referring clinicians also reported appreciation of having the additional resource of the quitline to assist with treating smoking patients and reported greater personal motivation related to the topic of tobacco cessation, which was not reported by the non-referring clinicians. Although the feedback provided through the randomized trial was mentioned as a reminder for referral to the quitline, it appeared to be only one factor of many that influenced the clinicians to refer to the quitline.

We found consistency in our findings with previous research. First, clinicians referring at a higher rate reported greater personal interest in the topic of tobacco cessation [20,21]. Second, patient factors such as readiness to quit or perceived resistance were mentioned, but did not play a major factor in influencing the referrals across the two groups in our study [22]. Last, the clinicians reported multiple barriers to smoking cessation intervention in primary care such as the time it takes, lack of reimbursement and competing demands [16,23]. The study participants highlighted, however, specific suggestions for overcoming these barriers including having one central referral source (eliminating the barrier of matching insurance status with program), having a referral coordinator, and having reimbursement for tobacco counseling as a billable code. Clinicians also reported a desire for referral sources to provide information on the success with cessation of each patient referred.

A question that arises from these data is whether clinicians feel, in regard to tobacco cessation, they should be paid to counsel or paid to refer to quitline services. Research suggests that clinicians may address tobacco with patients at a greater rate if consistently paid to do so [24,25]. Our data suggests that clinicians are most comfortable with reimbursement for the counseling portion of the visit, regardless of whether referral occurs or not. None of the clinicians advocated for being paid for each referral made (i.e. incentive payment). Not all clinicians are comfortable with simply identification and referral (such as proposed with the "Ask and Act" program by the American Academy of Family Physicians [26]; and not all patients will accept referral. Regardless of additional referral, the advice and counsel of the physician may be a motivator for many patients in supporting their efforts to change their tobacco use [27].

It is important to note that this qualitative study attempts to describe the strategies used and concerns of clinicians who were encouraged to refer their patients to a quitline. This type of analysis does not represent all clinicians and focuses on understanding the perceptions of the clinicians involved in this study for the purpose of considering options for future research and clinical practice. One limitation is that, although we do have the actual count of referrals to the quitline, determining that high-referrers were in fact referring at a high rate, the referrals to only one quitline were recorded. Capturing data on other mechanisms for referral may have provided a greater understanding of service needs of the participating clinicians. Clinicians were participants in a larger trial of a smoking cessation feedback intervention, indicating that they may have had previous interest in smoking cessation as a topic and may have been more motivated to participate in this study. However, many practices participated out of self-reported interest in research or educational connection with the study institution.

## Conclusion

The qualitative research method in this sub-study provided the opportunity to understand the story of each clinician with regard to using the 5 A's for tobacco treatment in everyday practice. As this type of research is best used for formative or evaluative research, a next step is to test specific suggestions for overcoming barriers, such as those provided in this investigation. Holtrop [28] has undertaken a study testing the use of a practice referral liaison to examine referrals to cessation services that includes patient-specific feedback to the clinician. Other investigations may include the use of a single referral source, payment for tobacco treatment counseling and referral, or pay for performance for smoking cessation interventions with patients. Additional research is needed to investigate these and other mechanisms to most effectively deliver smoking cessation interventions in and in conjunction with primary medical care.

## Competing interests

Three authors declare that they have no conflicts of interest (Holtrop, Malouin, Weismantel). Dr. Wadland has a license agreement with the health insurance company that provides the quit line described in this study. Dr. Wadland is the developer of the quit line program and receives a small annual license fee from the health insurance company, which is paid to Michigan State University Department of Family Medicine.

## Authors' contributions

JH completed the literature review for the manuscript. JH and RM coded and analyzed the interview data. DW conducted the selection of clinicians for interview. WW and DW conducted the quantitative data analysis to identify the practice and clinician types. All authors contributed to writing the manuscript and have read and approved the manuscript.

## Appendix

Semi-structured interview guide

1. Please describe your current procedure for identifying smoking patients. Please focus on what you do specifically, but also discuss what other providers in your practice do if they do it differently.

2. Please describe your current procedure for assessing if a smoking patient is interested in quitting. Please focus on what you do specifically, but also discuss what other providers in your practice do if they do it differently.

3. Please describe your current procedure for assisting smoking patients who wish to quit. Please focus on what you do specifically, but also discuss what other providers in your practice do if they do it differently.

4. How would you describe your level of satisfaction with your current procedure for identifying, assessing interest in quitting, and assisting with quitting for your smoking patients?

5. Please tell me about your experience with the quit line program from [health insurer].

6. Please tell me about your experience with other smoking cessation programs.

7. Which would you be more likely to refer to, if any, and why?

8. Describe your experience with the fax referral form for referring patients to the quit line program.

9. Describe your experience with the referral card to the quit line program that has the 1–800 number on it.

10. What were some of the barriers to implementing either of these methods?

11. How has this study affected your practice?

12. What motivates you to refer your patients to the quit line program?

13. Please describe any successful procedures that you may have heard about that other practices are using for referring patients to smoking cessation programs.

14. What are some of the barriers within your own practice for implementing these types of procedures?

15. INTERVENTION ONLY Describe your feelings about the feedback reports. How could these be more helpful to you?

16. Describe your feelings about giving incentives to primary care providers to do smoking cessation interventions with patients.

17. Do you have any questions about other preventive services to patients that you would like to see addressed by practice-based researchers? What specifically?

## Pre-publication history

The pre-publication history for this paper can be accessed here:


